# Understanding the Development of Self-Regulated Learning: An Intervention Study to Promote Self-Regulated Learning in Vocational Schools

**DOI:** 10.1007/s12186-022-09298-4

**Published:** 2022-09-09

**Authors:** Mathias Mejeh, Tanja Held

**Affiliations:** Department of Research in School and Instruction, Institute of Educational Sciences, Fabrikstrasse 8, CH-3012 Bern, Switzerland

**Keywords:** Self-regulated learning, Vocational school, Process analysis, Intervention study

## Abstract

Self-regulated learning (SRL) provides the foundation for building sustainable knowledge and is therefore important for schools, classrooms, and lifelong learning in general. Especially in vocational education and training, the concept of SRL remains fundamental as it relates to preparing future employees. However, further research is needed on how vocational students situationally regulate their learning process and the extent to which this may be related to a dispositional change in their SRL. In this study, we analyzed longitudinal questionnaire data from 159 students who attended either SRL-conducive or regular vocational classes. We refer to Perry and colleagues' (2018) framework of an SRL-conducive learning environment, which focuses on (meta)cognitive, motivational, and emotional aspects of learning. Using multilevel analysis, we found differences in the development of (meta)cognitive components of learning, whereas no clear differences could be identified for motivational and emotional components. The results support the assumption that process analyses can be used to draw a more differentiated picture of SRL in vocational schools. Moreover, indirect approaches to promoting SRL should be designed to include all SRL-relevant aspects.

## Introduction

Self-regulated learning (SRL) can be seen as a complex process including (meta)cognitive, motivational, emotional, and behavioral aspects, which also relates to social processes (Järvelä & Bannert, 2021). As such, SRL is understood as the autonomous, self-directed behavior of individuals who actively monitor and regulate goal-oriented actions to improve their knowledge and skills (Paris & Paris, 2001). SRL is relevant at all levels of education and is a prerequisite for lifelong learning (Alheit & Dausien, 2002; Baumeister, 2005; Bolhuis, 2003). In this context, vocational schools can be seen as important spaces preparing learners for lifelong learning in the workplace (Deissinger & Gonon, 2021). Therefore, the investigation of SRL in vocational schools is highly relevant from both a scientific and a practical perspective.

SRL has already been studied multiple times as an educational concept and has been excellently conceptualized from a theoretical point of view (see Panadero, 2017 for an overview). Empirically, a significant impact of SRL on academic performance (e.g., Cleary et al., 2020; Dignath & Büttner, 2008; Zimmerman & Bandura, 1994), well-being (e.g., Davis & Hadwin, 2021; Park et al., 2012; Zimmerman & Martinez-Pons, 1990), and the development of generic competencies (e.g., Artelt et al., 2001; Weinstein & Hume, 1998; Wolters, 2011) has been demonstrated.

Despite the extant literature, several aspects of SRL remain unexplored. An ongoing major issue is the question of student learning trajectories (Winne, 2019). Promising approaches have been developed to determine what exactly constitutes the learning trajectory (methodologically: e.g., Schmitz et al., 2012; content-related: e.g., Pintrich, 2000; Zimmerman, 2000). However, considering SRL as a process also raises the question of how individuals interact with their learning environment. Existing suggestions have focused on the evolution of learners’ SRL and change over time from a more externally determined to a more self-determined learning environment (for a related discussion, see Hmelo-Silver et al., 2007; Kirschner et al., 2006). Nevertheless, the role of the learning context can be seen as underrepresented in many studies on SRL and, therefore, not sufficiently investigated (Perry & Rahim, 2011). To address this issue, the goal of this paper is twofold: (1) to gain more detailed insight into students’ SRL trajectories in vocational schools and, in this context, (2) to demonstrate the importance of the learning environment for SRL. Of central importance are the constitutive elements for promoting SRL, rather than the outcome of SRL itself. In this paper, the results of an intervention focusing on key classroom features that foster SRL will be presented and discussed. Key classroom features represent a set of characteristics in the classroom context that emphasizes SRL, referring to student activities as much as teacher activities (Perry et al., 2018).

## Self-Regulated-Learning in Vocational Schools

Today, the promotion of SRL plays an important role as many companies expect their employees and learners to be self-directed and responsible learners (Dall'Alba, 2009; Ertl & Sloane, 2004; Kirschner & Stoyanov, 2018). For students to acquire these competencies, professional knowledge should also be developed through active and self-directed learning processes (Lang & Pätzold, 2006; Metzger et al., 2005). In this context, work-related SRL is a central factor in the professionalization process of aspiring employees. However, existing research indicates that the task orientation of vocational learners rarely takes place during hands-on simulations. Thus, the use of different (meta)cognitive strategies (e.g., time management, self-monitoring, or goal-setting) is, in many cases, not considered by vocational students (Khaled et al., 2015). Jossberger and colleagues (Jossberger et al., 2020), for their part, were able to show that, despite being able to effectively plan and monitor their self-regulatory activities, students are often unable to carry out their planned activities successfully.

At the same time, the promotion of SRL during vocational school lessons can be said to be very important as well, especially because it can be assumed that the number of learning opportunities in training companies will decrease in the coming years (e.g., due to megatrends) and that schools will play an increasingly important role in providing vocational education (OECD, 2021). Accordingly, a sustainable vocational education does not focus only on the acquisition of occupational skills but also on the development of generic competencies. The importance of vocational schools for the vocational training of learners is particularly evident in dual vocational training systems such as those found in Switzerland, Germany, Denmark, the Netherlands, or Austria. For example, a Swiss apprentice should not only learn to become a skilled worker for the labor market but also be able to continue learning at a higher level of the education system later on (Gonon, 2017). In a recent study, Kirschner and Stoyanov (2018) were able to show, based on a survey of experts, that SRL continues to play an important role for vocational learners in training because it forms the basis for lifelong learning. Correspondingly, the experts who were interviewed considered the promotion of cognitive and metacognitive strategies just as fundamental as learning in authentic learning situations. However, the learning context must be modified and adapted so that learners can develop their SRL competencies appropriately (Kirschner & Stoyanov, 2018). This shows that the development of job-specific competencies continues to play an important role, particularly because these are relevant for the transition from school to work. At the same time, successful SRL is also important for achieving and, more importantly, retaining employment (Forster-Heinzer et al., 2016; Hanushek et al., 2017).

SRL can be considered an essential prerequisite for lifelong learning, and the design of learning environments plays an exceptionally important role in effective SRL. In this context, instructional designs that link to learners’ competence development and shift over time from more externally to more self-directed instructions have been proposed several times (Dubs, 2015). The promotion of the different components of SRL (cognition, metacognition, emotion, and motivation) plays an equally important part. For example, in a series of studies on vocational education in Germany, Sembill and colleagues demonstrated that SRL-oriented instruction leads to higher learning motivation and problem-solving skills among vocational students while they develop the same degree of expert knowledge (Sembill, 1999; Sembill et al., 2001). In addition, SRL-conducive learning environments have been shown to lead to deeper interconnectedness across SRL phases (forethought, performance, and self-reflection), as well as significantly more questions from learners about the learning content and better feedback from teachers (Sembill, 2004). Accordingly, teachers should provide many opportunities for learners to participate in the classroom given that a greater experience of autonomy has a positive impact on vocational students’ motivation to learn and SRL (Sembill et al., 2001; Van Grinsven, 2003). This illustrates clearly for vocational education what is central at other levels of education as well: purposeful feedback and various forms of assessment of learning processes represent a fundamental prerequisite for SRL when they are intensively linked to the learning environment (Butler & Winne, 1995). However, in vocational education, these elements are only partially used (Rozendaal, 2002), resulting in limited recourse to metacognitive regulation strategies (van Velzen, 2004; van Velzen & Tillema, 2004).

Overall, vocational school instruction must be seen as a key component of the quality of training for vocational students, which should be subject to constant further development due to its lasting effects on the learning and action patterns of the students (Höpfer, 2017). This requires, inter alia, instructional measures in vocational schools through which young people can further develop their SRL competencies (Frey & Terhart, 2010; Sachs et al., 2016).

### Self-Regulated Learning and Learning Environment

Studies on the promotion of SRL have increasingly focused on the development of individuals based on the design of their learning environment, with fundamental importance given to teachers (e.g., Dignath & Büttner, 2008; Dignath et al., 2008; Kramarski, 2018; Kramarski et al., 2013; Waytens et al., 2002). This development is particularly necessary because it has been pointed out that not enough attention is paid to the interaction between the individual and the learning environment (Martin, 2007; McCaslin & Good, 1996; Perry & Rahim, 2011). In a recent review, various approaches to promote SRL have been provided (Dignath & Veenman, 2020). Based on this overview, direct promotion refers to teachers’ instruction of regulation strategies—further divided into explicit and implicit strategy instruction—, while indirect promotion refers to the design of a learning environment that fosters SRL. Direct strategy instruction is gradual in terms of its explicitness (e.g., Dignath & Büttner, 2008). Brown and colleagues (Brown et al., 1981) differentiate three different levels of direct strategy instruction in this regard. Whereas blind training refers only to the instruction of strategies without further contextual information on how to use them, informed training also provides students with information on the benefit of the given strategy. Self-control training combines strategies with explicit instructions on when, how, and where to use the provided strategies during the learning process (Dignath & Veenman, 2020). At this point, it becomes clear that teacher expertise is central to both direct and indirect promotion of SRL. According to Dignath and Veenman (2020), significant differences exist between teachers in terms of promoting SRL. For example, regulation strategies are prompted differently and often taught implicitly rather than explicitly (i.e., through verbalization). Furthermore, there is a positive relation between teachers' instruction of SRL strategies and students' use of them, while teachers' SRL beliefs are positively correlated to their SRL practice (Dignath & Veenman, 2020). Further research indicates that teacher self-regulation (e.g., Kramarski, 2008; Kramarski & Kohen, 2017), self-efficacy (e.g., De Smul et al., 2018; Dignath, 2016), motivation (e.g., Karlen et al., 2020) and knowledge of SRL (e.g., Spruce & Bol, 2015) are important predictors of learners' successful SRL. In this paper, the focus lies exclusively on the learning environment, that is, the indirect promotion of learning environments.

The relevance of learning environments for learning in general—and SRL in particular—has been widely demonstrated (Biggs, 1989, 1993; Boekaerts, 1992, 1996; De Corte, 1996; Entwistle, 1991; Vermunt, 1995; Vermunt & Donche, 2017; Zimmerman, 1989). In the school context, various instructional approaches have been developed since the 1980s, such as cognitive apprenticeship (Collins et al., 1989), situated learning (Greeno, 2006; Resnick, 1987), and problem-based learning (Barrows & Tamblyn, 1980). To promote SRL in school, some models explicitly include the learning environment. For example, the CLIA model (De Corte et al., 2004) emphasizes that the various components of competencies (**C**ompetence), characteristics of effective learning processes (**L**earning), principles and methods for designing a learning environment (**I**ntervention), and various forms of assessment (**A**ssessment) must be aligned to promote self-directed learning in students (De Corte et al., 2004, p. 368). Effectiveness studies revealed that students in SRL-supportive environments demonstrate more sophisticated mathematical problem-solving skills and have more positive attitudes and beliefs regarding mathematics (De Corte et al., 2004). In addition, more intense co-regulation between individual students (De Corte, 2012), higher achievement, and increased use of metacognitive regulation strategies have been demonstrated (De Corte, 2016; Masui & De Corte, 2005).

In line with this model, Perry and colleagues (Perry, 1998, 2013; Perry et al., 2018) developed a framework with different characteristics of the classroom context that emphasizes SRL. They summarize classroom characteristics in four macrocategories (“SRL-Supportive Structures,” “Student Influence and Autonomy,” “Supporting, Scaffolding, Co-Regulation,” and “Functions as a Community”), which, in turn, are subdivided into several microcategories that reflect the types of practices that teachers use. “SRL-Supportive Structures” are defined as (1) assigning meaningful tasks that are linked to clear instructions and expectations as well as (2) providing students with enough opportunities to participate in classroom activities. Particularly well-designed tasks lead to deeper information processing, more efficient use of regulatory strategies, and higher self-efficacy of the students (Perry, 2013). However, complex learning situations also require systematic and targeted support for learners (Reeve & Halusic, 2009). In this respect, Perry and colleagues (Perry et al., 2020a) were able to demonstrate that classrooms in which SRL is highly valued also provide structural support for SRL and the students’ autonomy. The macrocategory “Student Influence and Autonomy” refers to the availability of opportunities to co-design lessons and control one’s own learning, thus promoting student influence and autonomy. The selection, modification, and alteration of tasks, as well as various forms of self-assessment, are crucial to this process. Students who learn in autonomy-enhancing learning environments experience more positive emotions about their learning process, seek more challenging tasks (Su & Reeve, 2011), and show greater engagement, less amotivation (Cheon & Reeve, 2015), and increased autonomous motivation (De Naeghel et al., 2016). “Supporting, Scaffolding, Co-Regulation” and “Creating a Community of Learners,” as the third and fourth macrocategories, relate to the interactions between teachers and students as well as among the students themselves. Powerful learning environments are characterized by model learning, demonstration, metacognitive and motivational dialogue, and mutual and differentiated feedback (Perry et al., 2020b). In this regard, the importance of social interactions for SRL-enabling learning environments, which is discussed extensively in the context of socially shared regulation, is emphasized (Hadwin et al., 2018). The importance of the social context has been demonstrated several times, for example, regarding scaffolding through teachers and peers (Leeuwen & Janssen, 2019; Molenaar et al., 2014; Salonen et al., 2005; Winstone et al., 2016) or collaborative learning (McCaslin & Vriesema, 2018; Panadero et al., 2015; Vriesema & McCaslin, 2020). Thereby, positive effects regarding shared goal orientation (Isohätälä et al., 2017), performance (Janssen et al., 2012), and the quality of regulatory processes in groups (De Backer et al., 2015) can be distinguished. Furthermore, the relevance of group-regulated learning for productive cognitive interaction (Khosa & Volet, 2014), supportive socio-emotional interaction (Rogat & Adams- Wiggins, 2015; Rogat & Linnenbrink- Garcia, 2011), and even their interplay (Barron, 2003; Sinha et al., 2015) was demonstrated. So, when a classroom takes on a positive climate, characterized by shared knowledge, respectful communication, acknowledgment of individuality, and mutual support, the classroom functions as a community. It was found that establishing a community of learners is conducive to SRL because learners seek more help and support from each other more intensively overall (Perry & Drummond, 2002). Together, these categories summarize characteristics of the classroom context that emphasize students’ SRL (Perry et al., 2015) and serve as the theoretical foundation in this study (Perry et al., 2018).

However, it must also be noted that the manifestation of SRL depends not only on the learning context (Winne & Hadwin, 1998) but also on how regulation evolves over time (McCardle & Hadwin, 2015). On the one hand, there is the view that stable personality characteristics are decisive; on the other hand, there is the conviction that, above all, the current situation is crucial to appropriately analyze SRL. Therefore, time is an important component for the understanding of SRL (Patrick & Middleton, 2002).

### Self-Regulated Learning as a Temporal Process

Whereas in the early stages of its theoretical conceptualization, SRL was primarily defined as a disposition and empirical studies have measured it as a dispositional trait (Boekaerts & Corno, 2005; McCardle & Hadwin, 2015; Winne, 2019; Winne & Perry, 2000), contemporary views understand SRL as a dynamic and repetitive process in which the (meta)cognitive, emotional, and motivational components of learning (seen as states) unfold over time. Thus, the effective self-regulation of learning is fundamentally dynamic in various phases of learning and can be flexibly modified to suit the learning environment (and associated requirements) (Greene et al., 2021). If regulation is understood as action and/or behavior that develops over time, SRL can be seen as a series of events during a learning task, which should be captured and analyzed in terms of its process (McCardle & Hadwin, 2015; Winne, 2019). Learners who effectively self-regulate their learning set learning goals and continuously adjust their efforts by monitoring the achievement of their learning goal (Bernacki, 2018; Greene & Azevedo, 2010). This illustrates the relevance of metacognitive processes because SRL can be measured via concrete events in class, for instance, when students solve tasks or edit texts (Greene et al., 2021). In this context, different types of self-regulation can be identified and approached as different metacognitive processes, such as task understanding, elaboration, evaluation, and monitoring (McCardle & Hadwin, 2015). In their study, McCardle and Hadwin (2015) demonstrated that metacognitive awareness changes over time and has a significant influence on how learners control and organize their learning process. Nonetheless, other components of SRL also evolve. For example, Moos and Azevedo (2008) showed that not only (meta)cognitive components but also emotional and motivational components fluctuate during learning. The results of their study indicate that learners develop more sophisticated strategies over time to solve tasks, along with an increasing interest in the tasks themselves.

Viewing SRL as a dynamic process rather than a disposition has led to a great deal of discussion in recent years, in which the measurement of SRL processes is still considered a major challenge (Veenman et al., 2006; Winne, 2010). The need to measure SRL as a process, via so-called online measures, has been expressed several times (Molenaar & Järvelä, 2014; Winne & Perry, 2000; Zimmerman, 2008). Some innovative instruments such as think-aloud protocols (Sonnenberg & Bannert, 2019), log files (Bernacki, 2018), data mining (Lajoie et al., 2021), or electrodermal activity (Malmberg et al., 2019) have been developed in recent years. For example, Molenaar et al. (2021) have depicted student learning progress through moment-by-moment learning curves, thus providing deeper insights into when students need additional learning support.

Based on the foregoing, it is clear that SRL can be measured at different levels of granularity. Granularity refers to the level of detail at which self-regulatory processes are assessed (Azevedo, 2009). The decision to measure SRL finely or coarsely depends largely on the research question. Coarse-grained measures aim to capture the global process phases of learning (Rovers et al., 2019). Fine-grained measurement, in contrast, concerns the micro-level of learners’ SRL processes. An example is Schmitz and Wiese’s (2006) study, which examined the development of students’ learning over several days. Learning diaries were used for five weeks to track the development of self-regulatory behavior. Similarly, McCardle and Hadwin (2015) presented different types of self-regulation measured over 11 weeks by combining qualitative and quantitative methods. Several other studies have attempted even more fine-grained analysis of the SRL process. For instance, hypermedia learning sessions have revealed the relationship between cognitive and metacognitive processes during task solving (Azevedo et al., 2010). In a 60-min experiment (carried out in 10-min segments), it was shown that during task solving, learning strategies were used far more often (76.67%) than metacognitive strategies such as planning (4.80%) or monitoring (15.56%; Azevedo et al., 2010, p. 216). In studies on the dynamics of SRL, units of time are conceptualized differently, creating an artificial division. That is, time is segmented in various ways and can refer, for example, to individual lessons or entire teaching units over several weeks. It is therefore fundamental to link the segmentation of defined periods to clear guidelines and justify them theoretically (Molenaar, 2014). Although new and innovative methods have been developed in recent years for the measurement of SRL, there is still a lack of studies examining SRL as a process over time (Järvelä & Bannert, 2021). This can be noted in particular for vocational education and training, as “little is known about vocational students’ learning and their strategy use in real time” (Jossberger et al., 2020, p. 135).

## The Present Study

Vocational education provides a notably promising environment for the promotion of the development of SRL (OECD, 2021). Fostering SRL may require reforming the way that learning is organized and implemented in vocational schools. Physical spaces and new or adapted teaching materials are key factors in this process (Musset, 2019). At the same time, there is the question of whether students become more self-directed in dealing with different learning situations as they grow in age and experience (Boekaerts, 1996). Therefore, in this study, we designed an intervention that possesses some essential features of an SRL-conducive learning environment (for details, see “**Intervention**” section).

The overall aims of the present study were twofold. Our first goal was to investigate the development of SRL components over time. Due to the complexity of SRL, Pintrich’s model (2004) focuses on three areas of metacognition, motivation, and emotions, which remain very broad constructs. As such, the area of (meta)cognitive strategies is specified by the strategies of repetition, organization, elaboration, planning, monitoring, regulation, effort, time management, learning with fellow students, and learning environment. Motivational regulation is assessed by the two poles (intrinsic and external regulation) of Deci and Ryan’s (2002) continuum structure to reflect motivation within SRL. Finally, with regard to emotions, two common emotions (enjoyment and boredom) are examined that have different valences (positive and negative) and activation (activating and deactivating; Pekrun, 2006). All of these components are explicitly reflected in the framework of Perry et al. (2018), which forms the basis for our intervention.

Our second goal was to analyze whether an SRL-promoting learning environment in vocational schools may have an impact on students’ development of these SRL components in comparison with regular instruction. To date, too little is known about the process of SRL (Järvelä & Bannert, 2021; Winne, 2019). To better understand how SRL unfolds over time, it is therefore necessary to relate SRL as static competence (dispositional development) and SRL as strategic adaptation (situational development) to one another (McCardle & Hadwin, 2015). Thus, based on the different levels of granularity of SRL processes (Azevedo, 2009), we addressed this desideratum by analyzing the development of SRL components at two different measurement levels: the macro level to capture potential changes in students’ dispositions over a school year (coarse grained), and the meso level to examine weekly trends in SRL components over a semester (fine grained). The following research questions and hypotheses were investigated regarding the dispositional change and situational development of vocational students’ SRL components. To examine changes in students’ disposition in SRL components, our first research question is as follows:

Do dispositional changes in the use of (meta)cognitive strategies, perceived motivation, and emotions of students in treatment classes differ from those of students in control classes? (RQ1)

Based on the encouraging results of existing research (e.g., Sembill et al., 2007; Van Grinsven & Tillema, 2006), we hypothesized that students in the treatment classes increase their use of cognitive and metacognitive strategies compared to students in the control classes. Previous research demonstrated that the satisfaction of students’ basic psychological needs (need for autonomy, competence, and relatedness) predicts positive emotions and contributes to intrinsic motivation (De Naeghel et al., 2016; Isen & Reeve, 2005; Ryan & Deci, 2020). Thus, because the SRL-setting is also assumed to better fulfill students’ basic psychological needs (Perry et al., 2018), we assumed that students in the treatment classes exhibit an increase in intrinsic motivation and positive emotions. Several studies revealed that basic need satisfaction is associated with higher internalization of externally motivated activities and a decrease in negative emotions (Skinner et al., 2017; Vansteenkiste et al., 2020; Yu et al., 2016). Therefore, we expect a decrease in extrinsic motivation and negative emotions compared to the control classes. For the control classes, we did not expect any changes in the development of the SRL components over a school year. To gain deeper insights into the development of learners’ SRL, our second research question is as follows:

Does the situational development in the students’ use of (meta)cognitive strategies, perceived motivation, and emotions in treatment classes differ from those of students in control classes? (RQ2)

In line with the existing research (Sembill et al., 2008; Wild, 2001; Wild & Krapp, 1996), we hypothesized increasing linear development in the use of (meta)cognitive strategies, intrinsic motivation, and positive emotions in the treatment classes. A linear development of SRL (e.g., Leidinger & Perels, 2012; Schmitz & Wiese, 2006) or positive emotions (Goetz et al., 2013) could be demonstrated over a similar period of time. For motivation and engagement, Martin and colleagues (Martin et al., 2015) were able to identify a linear development between weeks, even if non-linear developments between single days have been detected as well.

At the same time, we expected a decrease in extrinsic motivation and negative emotions in the treatment classes. For the control classes, we did not expect any changes in the development of the SRL components over a semester.

## Method

### Participants and Data Collection

The purpose of the present study is to evaluate an SRL-supportive instructional setting within a quasi-experimental study. The quantitative sample consisted of 159 commercial apprentices in seven classes, with a mean age of 16.64 years (*SD* = 2.23 years) at the beginning of their first school year at vocational school “Wirtschafts- und Kaderschule Bern” in “ Bern, Switzerland.” Learners were assigned to the treatment classes on a voluntary basis, i.e. the vocational learners were informed by the school about the content of the SRL setting before they could decide for themselves whether they wanted to participate.

**Fig. 1:**
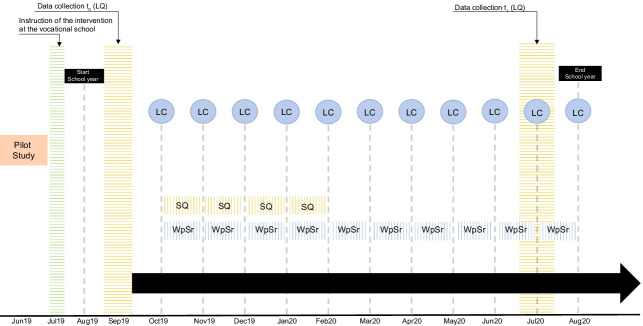
Research design. Note: LQ = Long Questionnaire (pre/post measurement); SQ = Short Questionnaire (intermediate measurement); LC = Learning Coaching; WpSr = Weekly planning/Self-reflection

Students in a vocational school in “ Switzerland” attend part-time classes two days a week and, on the other three days, are with their apprenticeship companies and attend no classes. Our intervention study only addresses learning in school and there is no transfer to the apprenticeship companies. Of these 159 students, 76 were male (47.8%) and 83 were female (52.2%). Three of the seven classes were intervention classes (*n* = 68; 42.8%), and four were control classes (*n* = 91; 57.2%). At the beginning (August) and end (June) of the school year, the students completed an extended online self-report questionnaire (Fig. 1). Between these long questionnaires, the students could participate in weekly short questionnaires during the school year. A total of 119 students (*n* = 46; 38.7% intervention vs. *n* = 73; 61.3% control) downloaded an application developed for this study onto their smartphones and participated in the weekly short questionnaire via this app. It took the students approximately one to two minutes to complete the short questionnaire. Once a week, the students received a push notification on their smartphones informing them that a new questionnaire was available. If they did not complete the questionnaire, the students received five more push notifications over the following days. To ensure that the results were not biased by a specific subject or day, a semi-randomized time interval was assigned for the data collection (Himmelstein et al., 2019). Data collection was tested with pilot studies. Before data collection, parental and student permission was obtained and the Ethics Committee of the Faculty of Human Science of the “University of Bern ” classified the study as safe/uncritical.

### Intervention

Our intervention approach refers to the framework model developed by Nancy Perry and colleagues, “Classroom Practices that Support Self-Regulated Learning” (Perry et al., 2018, 2020a). Based on this framework, we created a learning environment with different instructional elements to emphasize students’ SRL in a participatory approach with a vocational school (Perry et al., 2015). Table 1 illustrates which elements of the “Classroom Practices that Support Self-Regulated Learning” framework were included in our intervention.

**Table 1:** Classification of the content from the intervention into macro and micro categories (Perry et al., 2020b, p. 301f)

Macro Categories	Micro Categories	Instructional Setting (BGSOL)
Providing Structure	Tasks/Activities	Learning job/ 20 min Inputs
	Expectations/Instructions	Learning job/ 20 min Inputs
	Familiar Routines and Participation Structures	Learning job/ 20 min Inputs
	Visual Prompts	Learning job/ 20 min Inputs
Giving Students Influence	Involvement in Decision Making/Meaningful Choices	Weekly plans
	Control over Challenge	Weekly plans
	Self-Assessment	Self-tests
Supporting, Scaffolding, Co-regulation	Modeling/Demonstrating	20 min Inputs
	Questioning	Coaching
	Feedback	Self-tests
	Metacognitive Language	Coaching
	Motivational Messages	Coaching
Creating a Community of Learners	Co-constructing knowledge	Coaching
	Positive/Non-Threatening Communication	Coaching
	Supporting/Celebrating one Another’s Learning	Learning job
	Accommodations for Individual Differences	Learning job

In our intervention, a classroom structure was provided that allowed for autonomous learning (“SRL-Supportive Structures”). Most of the time, the students worked independently on individual assignments. For this purpose, they received a so-called “learning job” every four weeks. This document included all tasks to be completed, exam dates, and optional self-tests. At the same time, the learners received four to five 20-min input sessions during school hours, in which the technical content of the subjects Business and Society, Information, Communication, and Administration, German, English, and French were taught. During the school days, two teachers of different subjects were always available to answer students’ questions. In addition to the teachers’ input, exam times were also scheduled. Each week, an exam in one subject was held on the second day of school from 09:00 to 10:00. Students also had access to all documents and materials at any time via an online platform.

Regarding “Student Influence and Autonomy,” students in our intervention had the opportunity to design their learning process largely on their own. Based on their learning jobs, they created individual weekly plans and independently decided how much time they would spend on each subject at school and at home. The weekly plans served to control and assess SRL strategies and, thus, represented the central document to record the development of the vocational learners’ learning competencies. The main task of the students was to plan the processing of the learning assignments (in terms of time and content). With regard to self-assessment, they had the opportunity to check their learning progress via self-tests. The self-tests were formative and were coordinated with the teachers’ input.

Finally, “Supporting, Scaffolding, Co-Regulation” and “Creating a Community of Learners” relate to the interactions between teachers and students as well as among the students. In our intervention, these aspects were, inter alia, influenced by the coaching sessions. Each student was supported by a personal coach. At these coaching meetings, held every four weeks, in addition to discussing the self-tests and individual planning, various aspects of SRL such as applied learning strategies and time management were discussed, and individual goals were set. In addition, all tasks in the learning job could be completed in a chosen social form (e.g., partner or group work) to enable as much mutual support and co-regulation as possible.

Overall, the implementation of the intervention supported several macrocategories of Perry’s heuristic on “Classroom Practices that Support Self-Regulated Learning” and, thus, based on theoretical considerations, aimed to promote SRL among vocational students. The following approaches were used to evaluate implementation. Students’ weekly plans were evaluated by coaches and provided to the research team as a manipulation check. Additionally, the records of the coaching sessions were submitted to verify implementation. Finally, implementation of the inputs, learning jobs, weekly plans, self-tests, coaching sessions, and flexibility regarding social forms were verified through the interviews.

Besides the intervention setting, students in the control group attended regular classes with lessons in all subjects for 45 min each on both school days. After each lesson, they changed classrooms and teachers. Thus, the instructional design was primarily the responsibility of teachers and varied between subjects, and all instructional elements proposed to the students in the intervention setting (weekly plans, learning jobs, self tests) were not carried out in the control classes. In addition, there was no coaching or systematic individual mentoring in the regular classes.

### Measurement

As mentioned above, two types of measures were used for this study: a) long questionnaires at the beginning and end of the school year, and b) weekly short questionnaires. The long questionnaire consisted of 14 scales addressing the different components of SRL (Table 2). (Meta)cognitive strategies and resource management were measured using ten subscales: *repetition, organization*, *elaboration, planning*, *monitoring, regulation, effort, time management, learning with fellow students*, and *learning environment* of the “Inventory for the Measurement of Learning Strategies in Academic Studies” (LIST; Wild & Schiefele, 1994). Motivational components were measured using the *intrinsic* and *extrinsic motivation* components of the “German Self-Regulation Questionnaire” (Müller et al., 2007). Finally, for the emotional components, the two scales of *enjoyment* and *boredom* based on the German version of the “Achievement Emotion Questionnaire” (AEQ; Pekrun et al., 2005) were used.

**Table 2:** Overview of scales

Scale	Number of items	Example item	Cronbachs α within main questionnaire
Repetition^a^	7	I learn rules, technical terms or formulas from memory	α_t0_ = .70 α_t1_ = .84
Organization^a^	8	I write brief summaries of the main ideas to help me organize my thoughts	α_t0_ = .82 α_t1_ = .87
Elaboration^a^	9	I envision practical applications to new concepts	α_t0_ = .78 α_t1_ = .83
Planning^a^	8	I think about the order in which I study the material	α_t0_ = .79 α_t1_ = .89
Monitoring^a^	6	In my mind, I go back through the material I have learned to see if I have memorized all the essentials	α_t0_ = .66 α_t1_ = .82
Regulation^a^	6	If there is a difficult text, I adapt my learning technique to the higher demands	α_t0_ = .76 α_t1_ = .88
Effort^a^	6	I work until I am sure I can pass the exams very well	α_t0_ = .76 α_t1_ = .85
Time management^a^	4	When I study, I stick to a specific timeline	α_t0_ = .86 α_t1_ = .85
Learning with fellow students^a^	7	I collaborate on texts and assignments with my classmates	α_t0_ = .85 α_t1_ = .89
Learning environment^a^	6	When I study, I make sure that I can study in silence	α_t0_ = .77 α_t1_ = .82
Intrinsic motivation^b^	5	I work in vocational school because I want to learn new things	α_t0_ = .81 α_t1_ = .85
Extrinsic motivation^b^	6	I work in vocational school because otherwise I would get into trouble at my apprenticeship company	α_t0_ = .64 α_t1_ = .81
Enjoyment^c^	6	I enjoy vocational school	α_t0_ = .79 α_t1_ = .75
Boredom^c^	5	I get bored in vocational school	α_t0_ = .81 α_t1_ = .88

Likert scale range: ^a^ = 1 – 6 (never – always), adopted from Wild and Schiefele (1994); ^b^ = 1 – 5 (strongly disagree – strongly agree), adopted from Müller et al. (2007); ^c^ = 1 – 4 (is not true – is true), adopted from Pekrun et al. (2005)

The weekly short questionnaire consisted of one item of the scales of *repetition, organization, elaboration, planning, monitoring, regulation, effort, time management, learning with fellow students, learning environment, intrinsic motivation, extrinsic motivation, enjoyment,* as well as *boredom,* and was used over 14 weeks (one semester). Single-item measures have been reported to have adequate psychometric properties and represent a suitable alternative for long scales when those are not applicable (e.g., for frequent measures; Gogol et al., 2014). All single items were adopted from the long questionnaire (Table 2). For this purpose, we selected the items that best represented each corresponding scale (Goetz et al., 2013, p.387; Schmitz & Wiese, 2006). In the short questionnaire, all items were rated on a 4-point Likert scale ranging from 1 (not true) to 4 (true).

### Data Analysis

First, we wanted to investigate dispositional differences in the change of SRL components between students in the treatment and control classes. Linear mixed models have the advantage of allowing for the estimation of interindividual variability in intraindividual patterns of change over time (Raudenbush & Bryk, 2002). This allows estimation of a mean trajectory for the two groups, as well as a subject-specific difference for each individual (McNeish & Matta, 2018). We ran linear mixed-effect models based on data from the long questionnaires (start and end of the school year). This trait data corresponds to a nested data structure in which measures (Level 1; *N* = 318) are nested within persons (Level 2; *N* = 159). The number of missing values on the dependent variables ranged between 17% and 42.8%. This occurred as a result of the voluntary nature of participation in the study, absences due to illness during the survey period, and transfers from/to another school or profile within the school during the school year. Missing data in the long questionnaire were assessed with multiple imputations by a chained equation – package *mice* (van Buuren & Groothuis-Oudshoorn, 2011, version 3.13.0, number of imputed datasets m = 25 and iteration maxit = 25). Linear mixed models were run using the *lme4* package (version 1.1.27; Bates et al., 2015).

Second, to investigate the situational differences in the development of SRL components, we used data from the weekly short questionnaires (14 measurement points). This state data represents a nested data structure in which measures (Level 1; *N* = 1666) are nested within persons (Level 2; *N* = 119). The Table 6 in Appendix A shows variance components for all 14 variables. Due to the low group variances, a longitudinal two-level model was retained in our analyses (Level 1_time_ and Level 2_person_). Because of our intervention, we assumed a continuous development across time and, therefore, linear mixed-effect models were run using the *nlme* package (version 3.1.152; Pinheiro et al., 2021). Missing data were estimated using maximum likelihood estimates. All analyses were conducted in R (R Core Team, 2019).

## Results

Descriptive statistics and correlations for all the variables of the long questionnaire are presented in Table 3. In the first measurement, the two groups only differed in planning (*p* = 0.05). All other variables demonstrated no significant difference at t0. As expected, (meta)cognitive strategies and resource management were significantly correlated within measurement points. In addition, they usually correlated significantly with intrinsic motivation and enjoyment within measurement points. Interestingly, extrinsic motivation at the first measurement point was only significantly correlated with enjoyment, while significant correlations were found with (meta)cognitive strategies and resource management at the second measurement point. Boredom was significantly correlated with cognitive strategies, effort, learning environment, and enjoyment within the measurement points. All significant correlations showed the expected direction.

**Table 3:** Descriptive statistics and Pearson correlation

		*M (SD*)	TG	CG	1	2	3	4	5	6	7	8	9	10	11	12	13	14	15	16	17	18	19	20	21	22	23	24	25	26	27	28
			*M (SD*)	*M (SD)*																												
1	Repetition t0	3.93 (.69)	3.86 (.66)	4.03 (.74)	-	.31*	.39*	.23*	.17*	.15	.28*	.14	.33*	.21*	.35*	.19*	.34*	.18*	.21*	.10	.20*	.10	.29*	.16	.22*	.15	.12	.14	.19*	.10	-.16*	-.11
2	Repetition t1	4.06 (.86)	4.08 (.80)	4.02 (.88)		-	.17*	.56*	.10	.45*	.17	.42*	.17	.48*	.21*	.43*	.22*	.41*	.16	.22*	.07	.24*	.27*	.37*	.19*	.31*	.00	.25*	.13	.16*	-.20*	-.24*
3	Organization t0	4.14 (0.81)	3.96 (.77)	4.22 (.86)			-	.26*	.17*	.10	.45*	.12	.33*	.17	.36*	.13	.36*	.15	.18*	.12	.12	.04	.36*	.15	.23*	.08	.08	.02	.26*	.05	-.28*	-.10
4	Organization t1	4.10 (.94)	3.98 (.89)	4.19 (.98)				-	.02	.42*	.20*	.45*	.14	.49*	.20*	.44*	.21*	.43*	.22*	.28*	.13	.24*	.26*	.38*	.11	.29*	-.03	.17*	.08	.17*	-.17	-.20*
5	Elaboration t0	4.05 (.74)	4.00 (.76)	4.06 (.68)					-	.18*	.26*	.06	.27*	.13	.31*	.11	.20*	.08	.12	-.02	.07	.02	.09	.05	.25*	.09	.04	.07	.17*	.04	.17*	.04
6	Elaboration t1	3.94 (.81)	4.10 (.72)	3.82 (.81)						-	.13	.36*	.13	.42*	.16	.37*	.13	.32*	.09	.21*	.07	.27*	.22*	.29*	.18*	.37*	-.09	.16*	.11	.20*	-.10	-.09
7	Planning t0	4.05 (0.78)	3.88 (.74)	4.13 (.82)							-	.27*	.45*	.23*	.56*	.22*	.42*	.14	.44*	.18*	.24*	.08	.40*	.15*	.23*	.09	.10	.03	.17*	.09	-.15*	-.09
8	Planning t1	4.08 (0.92)	4.22 (.83)	4.00 (.94)								-	.10	.47*	.19*	.56*	.17	.36*	.29*	.30*	.07	.26*	.17	.34*	.15	.27*	-.14	.10	.05	.22*	-.15	-.11
9	Monitoring t0	3.75 (0.75)	3.72 (.67)	3.78 (.84)									-	.18*	.37*	.11	.38*	.18*	.27*	.15	.20*	.09	.23*	.14	.34*	.12	.06	.05	.27*	.14	-.12	-.07
10	Monitoring t1	3.89 (0.88)	3.88 (.81)	3.89 (.93)										-	.23*	.48*	.17	.42*	.20*	.26*	.16	.35*	.22	.33*	.17	.29*	-.03	.20*	.10	.21*	-.12	-.08
11	Regulation t0	4.52 (0.75)	4.42 (.76)	4.61 (.75)											-	.26*	.42*	.19*	.22*	.08	.10	.05	.37*	.21*	.26*	.13	.11	.08	.19*	.10	-.13	.03
12	Regulation t1	4.17 (0.95)	4.28 (.86)	4.11 (1.04)												-	.14	.38*	.23*	.20*	.10	.26*	.23*	.39*	.13	.27*	-.07	.19*	.06	.15	-.13	-.06
13	Effort t0	4.30 (.84)	4.27 (.84)	4.34 (.78)													-	.26*	.29*	.13	.01	.00	.33*	.24*	.40*	.19*	.09	.09	.39*	.15	-.38*	-.12
14	Effort1	4.24 (.81)	4.30 (.85)	4.22 (.86)														-	.18*	.34*	.01	.22*	.19*	.45*	.22*	.36*	-.01	.22*	.19*	.22*	-.24*	-.22*
15	Time management t0	3.13 (1.18)	3.13 (1.13)	3.12 (1.25)															-	.26*	.11	.08	.30*	.17	.20*	.07	-.05	.00	.12	.13	-.15*	-.11
16	Time management t1	3.56 (1.17)	3.52 (1.18)	3.63 (1.15)																-	.02	.18*	.12	.23*	.13	.25*	-.05	.04	.09	.23*	-.08	-.15
17	Learning with fellow students t0	4.00 (0.89)	3.92 (.90)	4.03 (.93)																	-	.27*	.03	.02	-.03	.01	.12	.03	.03	-.03	.06	-.12
18	Learning with fellow students t1	3.94 (1.00)	4.10 (1.00)	3.87 (1.01)																		-	.06	.19*	.04	.22*	.02	.15	.05	.17	.00	-.09
19	Learning environment t0	4.44 (0.77)	4.41 (.64)	4.52 (.88)																			-	.31*	.24*	.11	-.01	.05	.19*	.09	-.23*	-.15
20	Learning environment t1	4.41 (0.88)	4.49 (.79)	4.40 (.91)																				-	.15	.26*	-.09	.14	.17	.20*	-.23*	-.19*
21	Intrinsic motivation t0	3.14 (0.73)	3.21 (.65)	3.08 (.76)																					-	.28*	-.06	.01	.66*	.28*	-.34*	-.09
22	Intrinsic motivationt1	3.19 (0.79)	3.28 (.77)	3.06 (.75)																						-	-.04	.14	.27*	.42*	-.17	-.13
23	Extrinsic motivation t0	3.44 (0.63)	3.32 (.65)	3.52 (.63)																							-	.23*	.03	-.07	.00	.05
24	Extrinsic motivation t1	3.37 (0.78)	3.37 (.78)	3.37 (.79)																								-	.02	-.03	.01	.00
25	Enjoyment t0	2.36 (0.53)	2.40 (.49)	2.30 (.56)																									-	.26*	-.37*	-.16*
26	Enjoyment t1	2.44 (0.53)	2.51 (.52)	2.39 (.61)																										-	-.18*	-.08
27	Boredom t0	1.99 (0.58)	1.96 (.56)	1.99 (.61)																											-	.23*
28	Boredom t1	2.08 (0.65)	2.05 (.69)	2.11 (.66)																												-

TG = treatment group; CG = control group; *p ≤ .05

### Dispositional Change

To examine differences in the dispositional change of SRL components among students in treatment and control classes, separate linear mixed models were run from the long questionnaires to determine whether there was an interaction between the treatment and time (one school year).

The results revealed a significant interaction effect of time and the treatment in elaboration (*b* = -0.34, *t*(415.557) = -1.96, *p* = 0.05), planning (*b* = -0.48, *t*(182.251) = -2.37, *p* = 0.02), and learning with fellow students (*b* = -0.35, *t*(519.623) = -1.63, *p* = 0.10). Separate multilevel models revealed that time significantly predicted elaboration in the control group (*b* = -0.25, *t*(296.201) = -2.08, *p* = 0.04), whereas it did not in the treatment group (*b* = 0.10, *t*(432.930) = 0.74, *p* = 0.46). The interaction effect reflects the difference in slopes for time as a predictor of elaboration, meaning that elaboration in the students’ learning process decreased between the two measurement points in the control group, while it increased in the treatment group, although not significantly. In addition, separate multilevel models revealed that time significantly predicted planning in the treatment group (*b* = 0.34, *t*(453.040) = 2.53, *p* = 0.01) but not in the control group (*b* = -0.13, *t*(111.667) = -0.92, *p* = 0.36). The interaction effect, therefore, reflects the difference in slopes for time as a predictor of planning, such that the treatment group increased planning in their learning process between the two measurement points, while the control group lowered it, although not significantly. Separate multilevel models revealed that time did not significantly predict learning with fellow students in either the treatment (*b* = 0.18, *t*(157.227) = 1.01, *p* = 0.31) or the control group (*b* = -0.17, *t*(178.868) = -1.06, *p* = 0.29). Therefore, the interaction merely reflected the significant trend of the two groups as a whole. All other variables showed no significant interaction effects of time and the treatment (Table 4).

**Table 4:** Linear mixed-effect models based on data of the long questionnaires

	b	SE b	95% CI	p
Repetition				
Time	0.44	0.29	-0.03, 0.91	.13
Intervention	0.16	0.14	-0.06, 0.39	.24
Time x Intervention	-0.22	0.18	-0.52, 0.08	.22
Organization				
Time	.07	.36	-0.53, 0.67	.85
Intervention	.26	.15	0.00, 0.51	.10
Time x Intervention	-0.05	.21	-0.40, 0.30	.82
Elaboration				
Time	0.44	0.29	-0.03, 0.91	.13
Intervention	0.06	0.13	-0.16, 0.28	.64
Time x Intervention	-0.34	0.18	-0.63, -0.05	.05
Planning				
Time	0.82	0.31	0.30, 1.33	.01
Intervention	0.26	.15	0.02, 0.49	.08
Time x Intervention	-0.48	.20	-0.81, -0.14	.02
Monitoring				
Time	.22	.33	-0.32, 0.75	.51
Intervention	.06	.14	-0.17, 0.30	.65
Time x Intervention	-0.06	.19	-0.38, 0.26	.77
Regulation				
Time	0.23	.36	-0.37, 0.82	.53
Intervention	.19	.15	-0.05, 0.43	.19
Time x Intervention	-0.36	.22	-0.73, 0.01	.11
Effort				
Time	0.17	0.33	-0.37, 0.71	.60
Intervention	0.06	0.14	-0.17, 0.30	.67
Time x Intervention	-0.15	0.20	-0.48, 0.18	.46
Time management				
Time	0.29	0.45	-0.45, 1.03	.52
Intervention	-0.01	0.20	-0.34, 0.32	.97
Time x Intervention	0.11	0.27	-0.34, 0.56	.69
Learning with fellow students				
Time	.52	.36	-0.07, 1.12	.15
Intervention	.11	.17	-0.16, 0.38	.51
Time x Intervention	-0.35	.21	-0.69, 0.00	.10
Learning environment				
Time	.28	.29	-0.20, 0.76	.28
Intervention	.11	.14	-0.12, 0.34	.11
Time x Intervention	-.20	.18	-0.50, 0.09	.26
Intrinsic motivation				
Time	.17	.28	-0.29, 0.63	.54
Intervention	-.13	.13	-0.34, 0.09	.33
Time x Intervention	-.10	.17	-0.38, 0.18	.57
Extrinsic motivation				
Time	.26	.29	-0.22, 0.73	.37
Intervention	.20	.12	0.00, 0.41	.10
Time x Intervention	-0.20	.17	-0.48, 0.07	.23
Enjoyment				
Time	.11	.20	-0.21, 0.44	.36
Intervention	-0.11	.09	-0.26, 0.05	.20
Time x Intervention	-0.01	.12	-0.21, 0.19	.38
Boredom				
Time	.07	.27	-0.38, 0.51	.81
Intervention	.04	.11	-0.15, 0.22	.74
Time x Intervention	.02	.16	-0.24, 0.29	.89

To examine within-group differences over time, separate multilevel models were run. They revealed a significant main effect of time on regulation in the control group (*b* = -0.49, *t*(101.878) = -3.13, *p* = 0.002), whereas in the treatment group, time did not significantly predict regulation (*b* = -0.13, *t*(138.968) = -0.83, *p* = 0.41). Regarding time management, separate multilevel models revealed a significant main effect of time in the treatment group (*b* = 0.34, *t*(214.284) = 1.98, *p* = 0.05, as well as in the control group (*b* = 0.51, *t*(183.413) = 2.59, *p* = 0.01). Therefore, both groups significantly increased their time management between the two measurement points. All other variables showed no significant effect of time in either the treatment or the control group.

### Situational Development

To investigate whether the situational development of students’ SRL components in treatment classes differed from that of control classes over 14 measurement points, separate linear mixed models were run to determine whether there was an interaction between treatment and time. Significant effects are described below, and —non-significant effects are reported in Table 5.

**Table 5:** Linear mixed-effect models based on data of the weekly short questionnaires (14 measurement points)

	*b*	*SE b*	95% CI	*p*
Repetition				
Time	0.03	0.01	0.01, 0.05	.02
Intervention	0.26	0.14	-0.02, 0.53	.07
Time x Intervention	-0.03	0.02	-0.06, 0.00	.08
Organization				
Time	0.00	0.02	-0.03, .03	1.00
Intervention	0.32	0.19	-0.05, .69	.09
Time x Intervention	-0.01	0.02	-0.06, 0.03	.57
Elaboration				
Time	0.00	0.01	-0.02, 0.03	.75
Intervention	0.02	0.13	-0.24, 0.27	.90
Time x Intervention	-0.01	0.02	-0.05, 0.02	.51
Planning				
Time	0.02	0.01	-0.01, 0.04	.22
Intervention	0.20	0.16	-0.10, 0.51	.20
Time x Intervention	-0.04	0.02	-0.08, -0.01	.02
Monitoring				
Time	0.01	0.01	-0.01, 0.04	.25
Intervention	0.17	0.13	-0.10, 0.44	.19
Time x Intervention	-0.03	0.02	-0.07, .0.01	.08
Regulation				
Time	0.02	0.01	0.00, 0.04	.05
Intervention	0.07	0.13	-0.20, 0.33	.60
Time x Intervention	-0.03	0.02	-0.06, 0.00	.05
Effort management				
Time	0.00	0.01	-0.02, 0.02	.97
Intervention	-0.09	0.14	-0.37, 0.18	.51
Time x Intervention	-0.02	0.01	-0.04, 0.01	.26
Time management				
Time	0.02	0.01	-0.01, 0.04	.12
Intervention	-0.31	0.16	-0.63, 0.00	.05
Time x Intervention	-0.02	0.02	-0.05, 0.02	.32
Learning with fellow students				
Time	0.01	0.02	-0.02, 0.04	.45
Intervention	0.10	0.19	-0.27, 0.46	.60
Time x Intervention	-0.02	0.02	-0.06, 0.02	.38
Learning environment				
Time	0.01	0.01	-0.01, 0.03	.58
Intervention	0.26	0.12	-0.02, 0.50	.03
Time x Intervention	-0.04	0.02	-0.07, -0.01	.01
Intrinsic motivation				
Time	0.00	0.01	-0.02, 0.02	.91
Intervention	-0.11	0.14	-0.39, 0.17	.45
Time x Intervention	-0.01	0.01	-0.03, 0.01	.41
Extrinsic motivation				
Time	0.01	0.01	-0.1, 0.04	.30
Intervention	0.18	0.17	-0.15, 0.51	.27
Time x Intervention	-0.01	0.02	-0.04, 0.03	.60
Enjoyment				
Time	0.02	0.01	0.00, 0.04	.07
Intervention	-0.25	0.15	-0.54, 0.04	.09
Time x Intervention	-0.02	0.02	-0.04, 0.01	.31
Boredom				
Time	0.01	0.01	-0.02, 0.03	.66
Intervention	0.18	0.15	-0.11, 0.47	.22
Time x Intervention	-0.003	0.02	-0.03, 0.03	.83

**Fig. 2:**
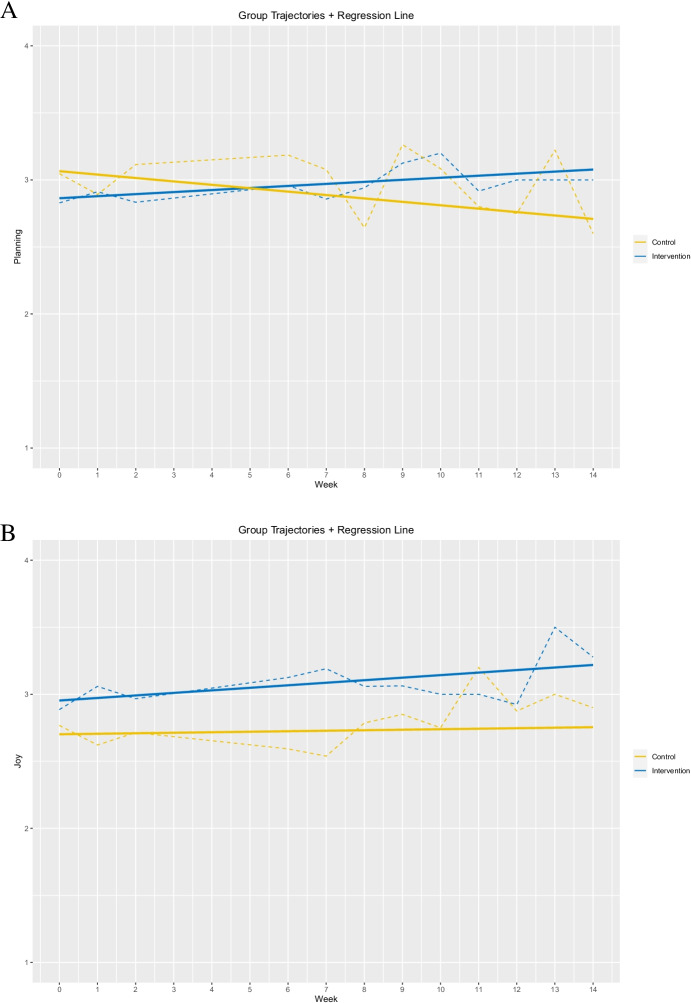
Group trajectories and regression line for planning (**A**) and joy (**B**) over 14 weeks. Note: Dash line = Group means; Solid line = Regression line

The results revealed a significant interaction effect of time and treatment in repetition (*b* = -0.03, *t*(407) = -1.74, *p* = 0.08), planning (*b* = -0.04, *t*(406) = -2.28, *p* = 0.05), monitoring (*b* = -0.03, *t*(408) = -1.67, *p* = 0.08), regulation (*b* = -0.03, *t*(408) = -1.93, *p* = 0.02), and structuring an appropriate learning environment (*b* = -0.04, *t*(409) = -2.73, *p* = 0.007). Separate multilevel models showed that time significantly positively predicted repetition in the treatment group (*b* = 0.03, *t*(206) = 2.51, *p* = 0.02), while no significant effect was found in the control group (*b* = -0.001, *t*(201) = -0.09, *p* = 0.92). Therefore, the interaction effect reflected the difference in slopes for time as a predictor of repetition. Repetition increased over the 14 weeks in the treatment group while remaining stable in the control group. Separate multilevel models indicated that planning was significantly negatively predicted by time in the control group (*b* = -0.02, *t*(200) = 1.98, *p* = 0.05). At the same time, it was not significantly predicted by time in the treatment group (*b* = 0.02, *t*(206) = -1.27, *p* = 0.20). The interaction effect reflected the difference in slopes for time as a predictor of planning, whereby planning decreased over the 14 weeks in the control group. Planning increased slightly in the treatment group, albeit not significantly (Fig. 2). In terms of monitoring, a separate multilevel model revealed that time was not a significant predictor for students in the treatment (*b* = 0.01, *t*(206) = 1.18, *p* = 0.24) or in the control group (*b* = -0.02, *t*(201) = -1.23, *p* = 0.22). Therefore, the interaction merely reflected the significant trend of the two groups as a whole. Regarding regulation, separate multilevel models revealed that time significantly positively predicted regulation in the treatment group (*b* = 0.02, *t*(207) = 1.98, *p* = 0.05), while no significant effect was found in the control group (*b* = -0.01, *t*(201) = -0.69, *p* = 0.49). This indicates that students in the treatment group significantly increased their regulation over time, whereas regulation remained stable in the control group. Finally, separate multilevel models showed that structuring an appropriate learning environment was significantly negatively predicted by time in the control group (*b* = -0.04, *t*(201) = -2.86, *p* = 0.005) but not in the treatment group (b = 0.01, *t*(208) = 0.52, *p* = 0.60). This indicates that the appropriate structuring of the learning environment by the learners in the control group decreased significantly over the 14 weeks but remained stable in the treatment group. All other variables showed no significant interaction effects of time and the treatment.

In addition, separate multilevel models brought to light a significant main effect of time on time management, learning with fellow students, and enjoyment. In terms of time management, the main effects of time were found for the treatment group (*b* = 0.02, *t*(208) = 1.70, *p* = 0.09), whereas in the control group, time did not significantly predict time management (*b* = -0.006, *t*(201) = -0.49, *p* = 0.62). A main effect of time on students’ learning with fellow students was found for those in the treatment group (*b* = 0.02, *t*(208) = 1.70, *p* = 0.09). For students in the control group, learning with others was not significantly predicted by time (*b* = -0.006, *t*(201) = -0.49, *p* = 0.76). Finally, a significant main effect of time was identified for enjoyment (Fig. 2): in the treatment group, enjoyment was significantly positively predicted by time (*b* = 0.02, *t*(205) = 1.87, *p* = 0.06), while no significant effect was found in the control group (*b* = 0.003, *t*(201) = 0.30, *p* = 0.76). These main effects indicate that students in the treatment group showed a significant increase in time management, learning with fellow students, and enjoyment. However, the effect cannot be clearly attributed to the intervention.

## Discussion

Today, understanding SRL as a process in its context is one of the key challenges in SRL research (Järvelä & Bannert, 2021; Winne, 2019). In this study, we analyzed questionnaire data from vocational students during their first year of study to investigate the development of the (meta)cognitive, emotional, and motivational components of learning over time. Moreover, we were interested in whether an intervention setting that aimed at fostering SRL in vocational education may change these developmental trends. To gain deeper insights, we investigated the dispositional change (RQ1) as well as the situational development (RQ2) of SRL components and looked for differences between the treatment and control groups.

### SRL as a Temporal Process

We found differences in dispositional changes in (meta)cognitive strategies like planning and elaboration within and between the two studied groups. There was also evidence of a situational development of (meta)cognitive variables like repetition, planning, and monitoring. As suggested, in the treatment group, the students’ disposition regarding planning increased significantly (Perry, 2013). In terms of elaboration and regulation, the results show that the intervention did not foster these strategies; instead, it was suggested that the intervention could protect students in the treatment group from a decrease. Therefore, based on the negative development of the control group, we noted the maintenance of elaboration and regulation as a positive result. For example, the significant negative effect of the trait measure over one year in the control group suggested that students spending more time in the normal school setting were less likely to use the cognitive strategy of elaboration, while this strategy remained constant in the treatment group. This is consistent with the findings of Bannert and colleagues (Bannert et al., 2014), who demonstrated that successful students use a cyclical sequence of SRL strategies that are repeated over time. Interestingly, in our study, both groups reported an increase in time management. Thus, this increase cannot be attributed to the intervention. Rather, we assume that the change in the school setting led to this increase. In compulsory education, students attend school from Monday to Friday, whereas in vocational school, they only spend two days in the school setting and the remaining three days in their training company. Accordingly, all students must adapt their time management to the new circumstances during their first year of vocational education (Wolters & Brady, 2020).

Our study showed that exploring the dispositional and situational attributes of SRL gives a better understanding of how vocational students learn in school (Sembill et al., 2008; Wild & Krapp, 1996). SRL is thereby seen as a process that becomes apparent through a series of events or actions in a certain temporal order (Molenaar & Järvelä, 2014). Against this backdrop, the weekly measurements helped to better understand the students’ general engagement in SRL. For instance, significant differences in the development of planning were observed between the two groups in both the trait and state measurements. However, separate models for each group revealed a significant decrease of the state measurement over time in the control group, while the trait measurement showed a significant increase in the treatment group. In addition, in the trait measurement, students in the treatment group reported lower levels of planning at the beginning of vocational education. This might indicate that students in the treatment group rated themselves lower in planning than the students in the control group because of the more complex setting. However, this increased complexity regarding students’ planning of their own learning could have had a long-term effect on students in the treatment group, resulting in a positive effect in the trait measurement. In contrast, students in the control group exhibited a decrease in planning in the state measurement because they became used to the new setting of only two school days and may have assumed that they must plan less than in lower secondary education (Xu et al., 2014). Thus, the decrease in planning in the control group during the first semester may be a transition effect that fades out with time. Equally important is the significant effect of regulation in the state measurement of the treatment group, reflecting an increase during the first semester, whereas in the trait measurement, no significant effect was found. This change could be explained by the strong situational variation of the variable, indicating that regulation varied strongly situationally but remained stable dispositionally (McCardle & Hadwin, 2015; Pintrich, 2004).

These results support the call for combined analyses of varying granularity (Rovers et al., 2019) and illustrate that intervention-based changes in SRL can be captured more sensitively by combining state and trait measures, while confirming findings from previous studies (e.g., Schmitz & Wiese, 2006). Moreover, the findings align with previous research in the area of SRL in the workplace (for an overview, see Cuyvers et al., 2020). For example, our results provide a complementary addition to the findings of Jossberger and colleagues (Jossberger et al., 2020), illustrating the contribution that vocational schools can make in promoting SRL and the extent to which this can be helpful in the workplace.

### SRL and the Learning Context

Concerning the striking development of the variables elaboration, structuring an appropriate learning environment, planning, repetition, and regulation in our study, our results are consistent with previous SRL research emphasizing metacognition for SRL (Bernacki, 2018; Greene et al., 2021). The particular relevance of metacognition for vocational learning has also been demonstrated (Kirschner & Stoyanov, 2018; Rozendaal, 2002; van Velzen, 2004). For instance, teachers who ask vocational students reflective questions influence their self-reflective thinking. Therefore, vocational students’ perceptions of teachers are particularly relevant, highlighting the importance of the relationship between teachers and learners (van Velzen & Tillema, 2004). However, in contrast to other (intervention) studies on SRL in vocational schools (e.g., Sembill et al., 2001), our intervention setting did not lead to increased positive emotions and intrinsic motivation. Although we assumed an indirect effect promoting SRL through our learning setting (Perry, 1998, 2013; Perry et al., 2018, 2020a), we could not identify any effects on the motivation variables, while only weak effects on enjoyment could be demonstrated. One possible explanation is that although a learning environment conducive to SRL was created in our study, no direct regulation strategies were taught; thus, the effects of combined strategy training are not attested, and the intended effect is missing (Paris & Paris, 2001). Finally, vocational students have been part of the school system for several years. Therefore, a stabilization of motivation and emotions over time (e.g., during adolescence; Gillet et al., 2012; Gläser-Zikuda et al., 2005) would also be possible, whereby motivation and emotions would have to be regarded more as a habitual pattern. Consequently, given the negative trajectories of motivation and emotion during primary and secondary education (Meyer & Schlesier, 2021; Raccanello et al., 2019; Scherrer & Preckel, 2019), a targeted promotion of these components would be important. To foster these aspects, the use of socio-psychological elements that explicitly focus on the meanings and inferences that students draw about themselves or situations has produced promising results (Walton & Wilson, 2018).

Overall, teacher competence on SRL could also be a decisive factor (Dignath & Veenman, 2020; Karlen et al., 2020). In our study, teachers' skills and attitudes towards SRL have not been included, but this could be an important explanation for the differential development in SRL. In addition, we assumed a moderation via basic psychological needs in our hypothesis, but this was not explicitly tested. It is possible that the SRL-conducive learning environment had no, little, or inconsistent influence on students’ perceptions of basic need satisfaction. At the same time, we treat the SRL of vocational students in this study independently of the subject content or tasks that learners were required to solve (Zimmerman, 2000). There is still little empirical research that intentionally explores this distinction (Alexander et al., 2011). However, researchers who distinguish between domain-specific (subject-related) and domain-nonspecific (subject-independent) SRL have also come to different conclusions in this respect. For example, Veenman & Spaans (2005) conclude that SRL changes gradually over time, with younger learners more likely to use domain-specific regulatory strategies and older learners more likely to demonstrate general SRL skills. In a recent study, metacognitive self-regulation strategies in digital learning environments were shown to be partly generic and partly domain-specific (Greene et al., 2015). Moreover, the role of the coaching sessions needs to be critically reflected: Although students should be holistically supported in their SRL through the coaching sessions, the coachings have mainly focused on (meta)cognitive aspects, such as planning or organization. However, this creates the risk that self-efficacy (Bandura, 1999; Hattie et al., 1996), for example, is not promoted as an important motivational component of SRL in a sustainable way.

### Limitations

Despite the advantages of the present study, notably its longitudinal approach over a school year and in-depth weekly measurements over a semester, some limitations must be taken into account. First, during the second semester, the COVID-19 pandemic affected our study. Because of the school’s closure, the students attended school from home for 12 weeks. This affected the treatment and control groups equally and may have impacted the results of the long questionnaire at the end of the school year, although the students were back at school at that time. In this context, the lockdown forced us to reduce our process analysis to 14 weeks (first semester), even though the measurement was originally planned for the entire school year. Thus, we cannot exclude the possibility of bias in the main and short questionnaires, and the results must be interpreted tentatively.

Second, because of the difference in granularity between the state and trait measurements, time is segmented in different ways (Azevedo, 2009), which might affect comparability between the two measurements and with other studies (e.g., Azevedo et al., 2010). In this study, fine- and coarse-grained measurements were related, and their temporal units were based on weekly measurements (situational development) and annual measurements (dispositional change). Thus, the determination of time units has a significant impact on how the results are interpreted (Molenaar, 2014). Third, the present study is exclusively based on quantitative self-reported data. The enrichment with qualitative data (e.g., think-aloud protocols; Sonnenberg & Bannert, 2019) and objective data (e.g., classroom observation, Dignath & Veenman, 2020; electrodermal activities, Malmberg et al., 2019) could provide deeper insight into the development of the SRL components of vocational students. Fourth, due to the large time span of the study and the number of weekly measurements, the number of missing values is high. Although modern techniques such as multiple-imputation and maximum likelihood estimations are appropriate to handle missing data (Buhi et al., 2008; Schlomer et al., 2010), the possibility of bias cannot be excluded.

### Implications and Future Research

The results of the present study regarding the effects of a structural SRL intervention in a vocational school provide important information for future research and practice. It can be assumed that the intervention positively affects students’ (meta)cognitive strategies. The effects on different levels of granularity, therefore, provide additional information on the overall impact of such an environment on students’ situational and lifelong learning (Kirschner & Stoyanov, 2018). For practice, the state measurement brought to light interesting individual trajectories of SRL components over time. Based on this data, researchers and/or teachers could react situationally to the developments of individual students and provide individual support (Molenaar et al., 2021; Reeve & Halusic, 2009). Given the increasing heterogeneity of students in classrooms, it is important in modern and future-oriented schools to focus not just on collective but also on individual learning paths.

In future studies, the structural intervention in vocational schools could also be aligned with an SRL intervention in training companies to achieve comprehensive promotion. In particular, this raises the question of how learners can be supported in their SRL by teachers (Karlen et al., 2020; Kramarski, 2018; Kramarski et al., 2013; Spruce & Bol, 2015). In doing so, it is important not only to create the appropriate environment for SRL but also to provide specific support for students in applying different strategies. Following the principle of scaffolding (Hmelo-Silver et al., 2007), teachers could, for example, use different forms of strategy instruction to support vocational learners individually, according to their stage of development (Dignath & Veenman, 2020). Thus, linking support via direct and indirect strategies would be an important direction for future intervention research on SRL (Paris & Paris, 2001).

Another important aspect relates to the implementation of the intervention: In our study, it became clear that, teachers implemented the coaching sessions differently despite being instructed to use a standardized manual. This in turn might have affected the heterogeneous results in the quantitative analysis. At this point, it becomes clear that the sustainable promotion of SRL among learners also always presupposes a structured learning environment and is not to be confused with minimal guidance (van Hout et al., 2000). Thus, effective implementation of instructional interventions is always a matter of instructional quality (Holtsch et al., 2019).

Finally, the possibility of aptitude-treatment interactions must always be considered when evaluating intervention research. The assumption is that the outcome of an intervention depends on the match between the students’ aptitudes and the treatment (Cronbach & Snow, 1977; Yeh, 2012). Thus, students differ in their readiness to profit from an intervention based on their individual aptitudes (Snow, 1992). Consequently, the intervention may not be equally effective for all students. Further research is needed to provide additional insight into whether there are systematic differences between students based on their aptitudes with respect to the effectiveness of the intervention.

## Data Availability

Data are available upon justified request after publication.

## Appendix

**Table 6:** Variance components for all variables (short questionnaire)

	Variance level 1 – time	Variance level 2 – person	Variance level 3 – group
Repetition	0.55	0.22	0.00
Organization	0.56	0.43	0.00
Elaboration	0.49	0.21	0.00
Planning	0.53	0.35	0.00
Monitoring	0.47	0.18	0.00
Regulation	0.37	0.28	0.00
Effort	0.36	0.25	0.00
Time management	0.47	0.41	0.02
Learning with fellow students	0.52	0.48	0.00
Learning environment	0.30	0.25	0.00
Intrinsic motivation	0.30	0.29	0.00
Extrinsic motivation	0.46	0.45	0.00
Enjoyment	0.25	0.48	0.01
Boredom	0.32	0.37	0.00

## Code Availability

Our R codes are available on request.
